# A Single Arm Pilot Study of Effects of Berberine on the Menstrual Pattern, Ovulation Rate, Hormonal and Metabolic Profiles in Anovulatory Chinese Women with Polycystic Ovary Syndrome

**DOI:** 10.1371/journal.pone.0144072

**Published:** 2015-12-08

**Authors:** Lin Li, Chengyan Li, Ping Pan, Xiaoli Chen, Xiaoke Wu, Ernest Hung Yu Ng, Dongzi Yang

**Affiliations:** 1 Department of Obstetrics and Gynecology, Sun Yat-sen Memorial Hospital, Guangzhou, China; 2 Department of Obstetric and Gynecology, Affiliated Hospital of Guangdong Medical College, Zhanjiang, China; 3 Department of Obstetrics and Gynecology, First Affiliated Hospital of Heilongjiang University of Chinese Medicine, Harbin, China; 4 Department of Obstetrics and Gynecology, University of Hong Kong, Hong Kong, China; Stavanger University Hospital, NORWAY

## Abstract

**Objective:**

To evaluate the effects of berberine on the menstrual pattern, ovulation rate, hormonal and metabolic profiles in anovulatory Chinese women with polycystic ovary syndrome.

**Methods:**

Berberine 0.4 g three times per day was given for four months to 102 anovulatory Chinese women with polycystic ovary syndrome. The menstrual pattern, ovulation rate, hormonal and metabolic profiles were compared before and after the berberine treatment. Ovulation was confirmed by serum progesterone level ≥10ng/ml.

**Results:**

A total of 98 of 102 subjects (96.1%) completed the four month treatment, including 69 (70.4%, 69/98) normal weight and 29 (29.6%, 29/98) overweight/obese. Fourteen women (14.3%, 14/98) had regained regular menses after berberine treatment and there was no significant difference between normal weight and overweight/obese groups. The ovulation rate was 25.0% over four months in the whole group, 22.5% in the normal weight group and 31.0% in the overweight/obese group. Sex hormone binding globulin, insulin resistance, total cholesterol, total triglyceride and low-density lipoprotein cholesterol decreased after berberine treatment in the normal weight group only.

**Conclusions:**

Our study found that administration of berberine alone may improve the menstrual pattern and ovulation rate in anovulatory Chinese women with polycystic ovary syndrome. Berberine can also decrease sex hormone binding globulin, insulin resistance, total cholesterol, triglycerides and low-density lipoprotein cholesterol in normal weight polycystic ovary syndrome women.

**Trial Registration:**

Chictr.org ChiCTR-OO-13003943

## Introduction

Polycystic ovary syndrome (PCOS) is one of the commonest disorders in reproductive endocrinology among women in the reproductive age and is diagnosed by the presence of two out of the following three: oligo- and/or anovulation, clinical and/or biochemical hyperandrogenism, and ultrasound features of polycystic ovaries, with the exclusion of other aetiologies [1]. Insulin resistance assessed by fasting glucose/ insulin ratio (GIR), homeostatic model assessment of insulin resistance (HOMA-IR) or quantitative insulin sensitivity check index (QUICKI) is a well recognised metabolic disturbance of PCOS [2]. It is found that insulin-sensitising agents can ameliorate the insulin responsiveness and the compensatory hyperinsulinaemia leading to improving ovulatory function.

Metformin, a biguanide, is the insulin-sensitising agent that is most commonly used in patients with PCOS. A Cochrane review showed that when compared with placebo or no treatment, metformin can improve the ovulation rate (OR 1.81; 95% CI 1.13–2.93) and clinical pregnancy rate (OR 2.31; 95% CI 1.52–3.51) but not the live birth rate (OR 1.8; 95% CI 0.52–6.16) [3]. Metformin has lower ovulation rate (OR 0.43; 95% CI 0.36–0.51), clinical pregnancy rate (OR 0.34; 95% CI 0.21–0.55) and live birth rate (OR 0.3; 95% CI 0.17–0.52) than clomiphene citrate (CC). Its common side effects include nausea, vomiting.

Berberine, a natural plant alkaloid, is isolated from the Chinese herb, Coptis Chinensis (Huanglian). It is usually used for diarrhea in China, and found to have a potential glucose lowering effect in recent years [4]. It has been used in PCOS women and may increase the success of in vitro fertilization (IVF) following improvement of insulin resistance. Li *et al*. reported that berbeirine 0.5g three times daily for 3 months in 120 PCOS women improved insulin resistance [5]. In a prospective study of 150 infertile PCOS women, 3 months of berberine treatment prior to an IVF cycle increased the live birth rate (48.6% vs 20.6%) compared with placebo [6]. There is still no published study on the use of berberine alone on the menstrual pattern, ovulation rate, hormonal and metabolic profiles of PCOS women.

The objectives of this prospective study were to evaluate the effects of berberine on the menstrual pattern, ovulation rate, hormonal and metabolic profiles in anovulatory Chinese PCOS women.

## Materials and Methods

### Subjects

This was a prospective study of 102 PCOS women recruited in the outpatient department of Gynecology in Sun Yat-sen Memorial Hospital from July 2012 to February 2013. The inclusion criteria were (i) Chinese; (ii) age >18 and <40 years; (iii) oligomenorrhea or anovulation (iv) clinical and/or biochemical hyperandrogenism or ultrasound features of polycystic ovaries; v) no fertility wish within one year. Those on hormonal preparation within 3 months were excluded. Subjects were divided into two groups according to their body mass index (BMI): (1) normal weight group: BMI < 23 kg/m^2^ and (2) overweight/obese group: BMI ≥ 23 kg/m^2^ [7]. This study had been approved by the ethic committee of Sun Yat-sen Memorial hospital and was registered in the Chinese Clinical Trial Register (ChiCTR) with the registration number of ChiCTR-OO-13003943. The purpose, procedure and potential risk of this study have been explained to each participant. Written informed consent was obtained before beginning the study.

### Study protocol

Berberine hydrochaloride tablets (0.1g/tablet, Guangdong Huanan Pharmaceutical Group Co. Ltd.) was given at 0.4 g three times daily for four months. Berberine was delivered to the participants individually every month. Menstrual calendar and side effect diary were recorded. Serum progesterone (P) and human chorionic gonadotropin (HCG) concentration were measured weekly to confirm ovulation and exclude pregnancy. After 4 months of berberine, the above assessments were repeated.

The primary outcomes were improvement of menstrual pattern and ovulation rates. The secondary outcomes were improvement of hormonal and metabolic profiles. Our original aim was to observe the subsequent effect of berberine for another 4 months after withdrawal. But our results demonstrated berberine had no effect on hyperandrogenism during the 4-month treatment. Depending on our results during the berberine treatment, we stopped the observation concerning the health of patients. The study ended in June 2013 after completing four-month follow-up.

The menstrual cycles were defined as following patterns: (1) regular menses with an intermentrual interval of 21–35 days; (2) oligomenorrhea with an intermenstrual interval > 35 days but < 6 months; (3) amenorrhea with an intermenstrual interval > 6 months; (4) irregular menses with an intermenstrual interval of 20–40 days and menses of 5 to 15 days [8]. Ovulation was confirmed by serum progesterone level ≥ 10ng/ml. Ovulation rate was calculated as the percentage of ovulatory cycles per total cycles [9].

Weight and height were measured before they joined the study. Hirsutism was assessed using the Ferriman-Gallwey hirsutism score and is defined as modified F-G score ≥ 5 [10]. After an overnight fast of at least 12 hours, the subjects underwent a transvaginal or transrectal ultrasound examination and a blood test on Day 2–5 of a spontaneous period or after a withdrawal bleeding. All ultrasound examinations were performed at 8–10 a.m. with a 5–9 MHz transvaginal probe of Shimadzu SDM-450 real time (Shimadzu Corp, Kyoto, Japan), after emptying the bladder. Fasting blood was taken for the measurement of follicle stimulating hormone (FSH), luteinizing hormone (LH), prolactin (PRL), estradiol (E_2_), total testosterone (TT), free testosterone (FT), sex hormone-binding globulin (SHBG), dehydroepiandrosterone sulphate (DHEAS), fasting plasma glucose, fasting insulin and lipid profile. The lipid profile included total cholesterol (TC), triglycerides (TG), low-density lipoprotein cholesterol (LDL-C) and high-density lipoprotein cholesterol (HDL-C). Free androgen index (FAI) is calculated by the equation: FAI = (TT (nmol/L)× 100)/SHBG (nmol/L) [11]. Homeostasis model assessment (HOMA) is calculated by the equation: HOMA-IR = (fasting plasma glucose (mmol/l) × insulin (IU/ml))/22.5. Insulin resistance (IR) was defined as HOMA ≥ 2.14 [12].

### Measurements

FSH, LH, PRL, E_2_, TT, P and HCG were measured by the chemilumniescence immunoassays Access 2 (Beckman, Fullerton, CA). FT and SHBG were measured by the Access 2 ELISA kits (Beckamn, Fullerton, CA). Insulin was measured bychemiluminescencce immunoassays (Immulite 200 Analyzer, CPC, Los Angeles, CA). Glucose was measured using the glucose oxidase and TC, TG, LDL-C, HDL-C were measured using an enzymatic calorimetric method with the 7600 antoanalyzer (Hitachi 7600, Tokyo, Japan). The inter- and intra-assay variation were 3.5% and 5.6% for FSH, 3.6% AND 4.3% for LH, 1.42% and 3.32% for PRL, 12% and 15% for E2, 7.25% and 6.59% for P, 0.15% and 2.15% for HCG, 1.67% and 4.78% for TT, ≤10% and ≤10% for FT, 4.0% and 5.3% for SHBG, 1.44% and <6% for glucose, 2.59% and <6% for TG, 2.11% and <6% for TC, 2.22% and <6% for HDL-C, 2.08% and <6% for LDL-C.

### Statistical analyses

The primary analysis was intention to treat and included all subjects with available 4-month outcome data. Normal-distributed continuous variables were given as mean ± standard deviation (SD). Non-normal-distributed continuous variables were given as median (inter quartile range). Continuous variables were compared by Student’s T test or Mann-Whitney U-test. Categorical variables were compared by Chi-square test or Fisher’s Exact Test. Statistical analysis was carried out using the Statistical Program for Social Sciences (SPSS Inc., Version 18.0, Chicago, USA). Statistical significance was set at a *P* value less than 0.05. Subgroup analysis was carried out according to normal weight and overweight/obese groups.

## Results

### Study group

**Fig 1:**
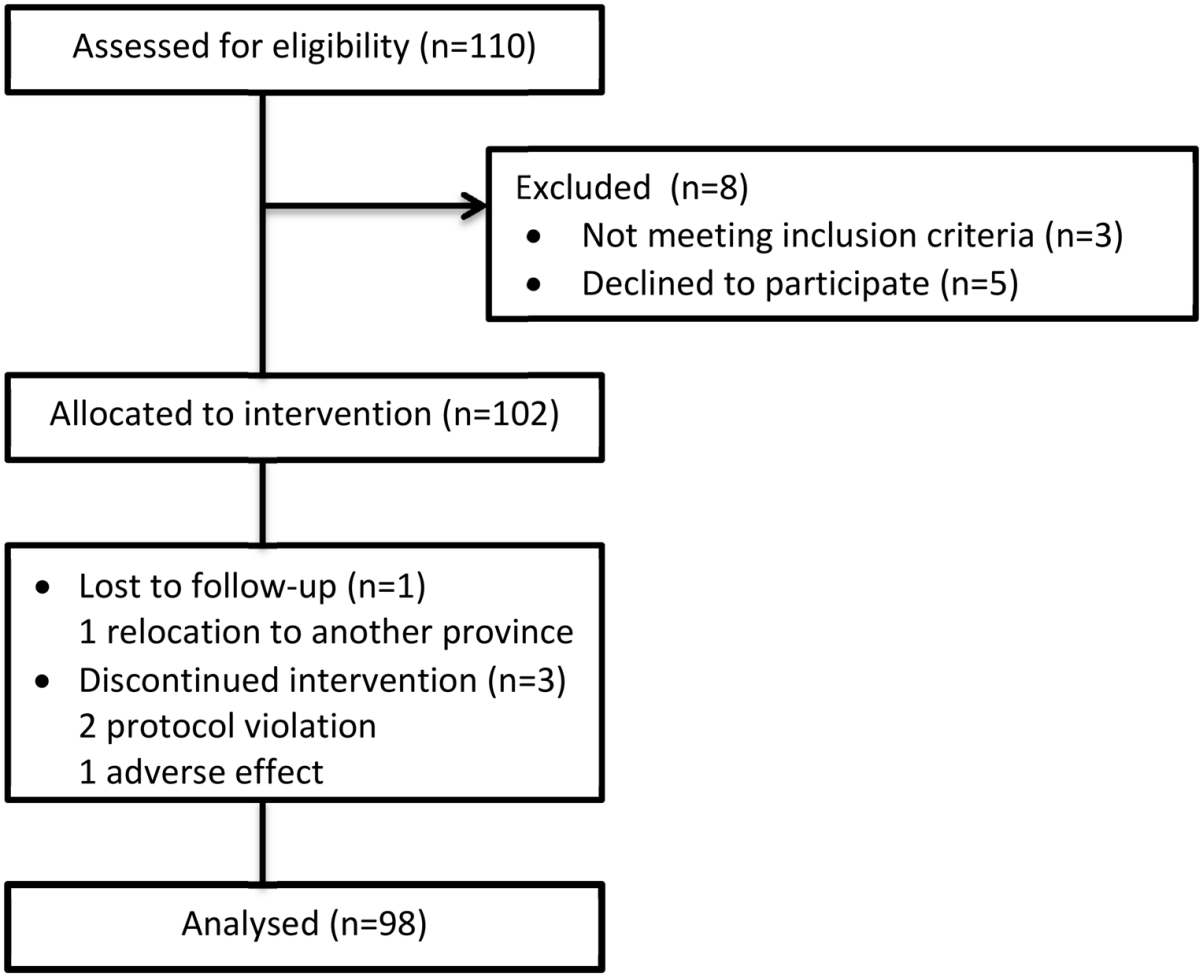
Enrollment and outcomes.

A total of 98 of 102 women (96.1%) completed the whole study period with full dataset. 2 women stopped the treatment, 1 lost to follow-up, and another for gastrointestinal side effects (Fig 1). All other subjects tolerated the treatment well without serious complaints or side effects. The median age of 98 women was 22 (18–39) years. The median BMI was 21.3 (15.6–32.1) kg/m^2^. 29 women (29.6%, 29/98) were overweight/obese i.e. BMI ≥ 23kg/m^2^ while 15 patients (15.3%, 15/98) were obese i.e. BMI ≥ 25kg/m^2^. A total of 59 women (60.2%, 59/98) had hirsutism and 51 (52.0%, 51/98) had acne.

### Effect on the menstrual pattern

The menstrual pattern at baseline was shown in Table 1. The menstrual pattern after treatment was significantly different from that before treatment in the whole group (*P*<0.001) and 14 women (14.3%, 13/98) had regained regular menses after treatment (11 in the normal weight group and 3 in the overweight/obese group). No significant differences were found in the menstrual pattern both before and after treatment between normal weight and overweight/obese groups.

**Table 1:** The menstrual pattern before and after 4-months of berberine in normal weight and overweight/obese groups.

Menstrual pattern	Whole group (n = 98)		Normal weight (n = 69)		Overweight/obese (n = 29)	
	Pre-treatment	Post-treatment	Pre-treatment	Post-treatment	Pre-treatment	Post-treatment
Regular menses (%)	0	14(14.3%)**	0	11 (15.9%)**	0	3 (10.3%)*
Oligomenorrhea (%)	74 (75.5%)	75 (76.5%)	50 (72.5%)	50 (72.5%)	24 (82.8%)	25 (86.2%)
Amenorrhea (%)	9 (9.2%)	3 (3.1%)	7 (10.1%)	3 (4.3%)	2 (6.9%)	0
Irregular menses (%)	15 (15.3%)	6 (6.1%)	12 (17.4%)	5 (7.2%)	3 (10.3%)	1 (3.4%)

*P<0.05,

**P<0.01, pre-treatment *vs* post-treatment.

### Effect on the ovulation rate

**Fig 2:**
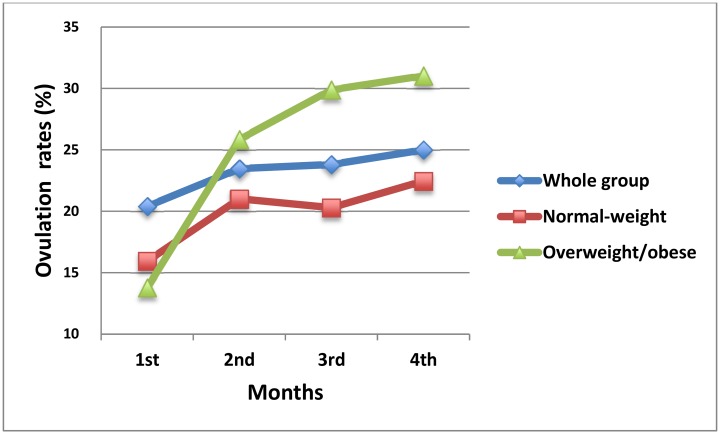
The ovulation rates during the 4-month berberine treatment. The ovulation rates (no. ovulatory cycles/no. cycles (%)) during the 4-month berberine treatment in the whole group, normal-weight group and overweight/obese group.

Fifty-one (52.0%, 51/98) women had at least one ovulation during the treatment period: 26 (26.5%, 26/98) had one ovulation, 11 (11.2%, 11/98) had two ovulations, 6 (6.1%, 6/98) had three ovulations, 8 (8.1%, 8/98) had four ovulations. The ovulation rate was 22.5% and 31.0% after 4-month treatment in the normal weight and overweight/obese groups, respectively. (Fig 2 and Table 2).

**Table 2:** Ovulation rates during the 4-month berberine treatment.

Month	Ovulation rate (no. ovulatory cycles/no. cycles (%))			*P* value
	Total	Normal weight	Overweight/obese	
**1** ^**st**^	15/98 (15.3%)	11/69 (15.9%)	4/29 (13.8%)	0.79
**2** ^**nd**^	43/196 (21.9%)	28/138 (21.0%)	15/58 (25.9%)	0.39
**3** ^**rd**^	67/294 (22.8%)	42/207 (20.3%)	25/87 (29.9%)	0.11
**4** ^**th**^	98/392 (25.0%)	62/276 (22.5%)	36/116 (31.0%)	0.07

Normal weight *vs* Overweight/obese.

### Effect on hormonal and metabolic profiles

The SHBG level decreased after berberine treatment in the whole group and such reduction was observed in the normal weight group only. No difference was found in TT, FT, fasting insulin and HOMA-IR. The incidence of insulin resistance decreased in the whole group and normal-weight group. Compared with baseline, TC, TG and LDL-C decreased after treatment in the whole group and such reduction was again found in the normal weight group only. Only TC decreased in the overweight/obese group after treatment (Tables 3 and 4).

**Table 3:** Hormonal and metabolic parameters before and after berberine in the whole group.

Variables	Pre-treatment	Post-treatment	*P* value
**TT (nmol/L)**	2.24(1.01–6.12)	2.03(0.32–3.98)	0.44
**FT (pg/mL)**	3.56(1.13–13.07)	3.28(1.03–10.47)	0.64
**DHEAS (ng/mL)**	2011.91(403.58–4261.00)	2141.00(752.41–4334.00)	1.00
**SHBG (nmol/L)**	61.03(12.29–203.04)*	35.16(8.04–157.09)*	0.02
**FAI**	3.97(0.50–33.16)	5.34(0.92–22.07)	0.29
**Fasting glucose (mmol/L)**	4.80(3.80–6.30)	4.80(4.10–5.60)	0.87
**Fasting insulin (mU/L)**	13.25(3.41–40.61)	11.65(5.48–40.85)	0.26
**HOMA-IR**	2.86(0.65–9.50)	2.44(1.19–6.44)	0.45
**IR (%)**	65.3	56.1	<0.001
**TC(mmol/L)**	4.86(3.21–6.97)*	4.03(2.70–6.53)*	0.002
**TG (mmol/L)**	1.12(0.26–3.55)*	0.70(0.35–2.38)*	0.002
**HDL-C (mmol/L)**	1.51(0.76–3.18)	1.35(0.68–2.44)	0.13
**LDL-C (mmol/L)**	2.72(0.96–5.36)*	2.40(1.24–3.92)*	0.001

Data are presented as Median (range); total testosterone;

FT, free testosterone;

DHEAS, dehydroepiandrosterone sulphate;

SHBG, sex hormone-binding globulin;

FAI, free androgen index;

HOMA-IR, homeostasis model assessment for insulin resistance;

IR, insulin resistance, HOMA≥ 2.14;

TC, total cholesterol;

TG, triglycerides;

HDL-C, high-density lipoprotein cholesterol;

LDL-C, low-density lipoprotein cholesterol.

*P<0.05, pre-treatment *vs* post-treatment.

**Table 4:** Hormonal and metabolic parameters before and after berberine in normal weight and overweight/obese groups.

Variables	Normal weight (n = 69)		Overweight/obese (n = 29)	
	Pre-treatment	Post-treatment	Pre-treatment	Post-treatment
**TT (nmol/L)**	2.29(1.01–6.12)	1.99(0.76–3.98)	1.99(1.14–5.41)	2.27(0.32–3.41)
**FT (pg/mL)**	3.36(1.13–13.07)	2.76(1.03–10.47)	4.07(1.38–7.89)	4.60(1.35–9.27)
**DHEAS (ng/mL)**	1975.00(403.58–4261.00)	1959.00(752.41.00–3568.00)	2048.00(1040.00–3693.18)	2209.00(1161.00–4334.00)
**SHBG (nmol/L)**	69.53(12.29–203.04)*	39.46(8.04–157.09)*	34.19(15.02–202.31)	26.13(8.30–54.85)
**FAI**	3.44(0.50–22.14)	4.40(0.92–16.30)	6.42(1.19–33.16)	6.96(4.22–22.07)
**Fasting glucose (mmol/L)**	4.80(4.07–5.80)	4.80(4.10–5.50)	4.90(3.80–6.30)	4.90(4.10–5.60)
**Fasting insulin (mU/L)**	10.67(3.41–36.33)	10.58(5.48–26.35)	20.51(9.21–40.61)	17.91(6.99–40.85)
**HOMA-IR**	2.25(0.65–8.07)	2.35(1.19–5.39)	4.71(1.56–9.50)	3.86(1.49–6.44)
**IR (%)**	53.6	44.9**	93.1	82.8
**TC(mmol/L)**	4.78(3.22–6.97)*	3.92(2.70–6.53)*	4.97(3.21–6.97)	4.19(3.22–5.21)*
**TG (mmol/L)**	0.93(0.26–3.49)**	0.68(0.35–1.73)**	1.18(0.64–3.55)	1.27(0.41–2.38)
**HDL-C (mmol/L)**	1.63(0.76–3.18)	1.43(1.02–2.44)	1.23(0.87–1.77)	1.24(0.68–1.55)
**LDL-C (mmol/L)**	2.63(0.96–5.36)**	2.37(1.24–3.92)**	3.13(2.02–4.85)	2.51(1.92–3.43)

Data are presented as Median (range); total testosterone;

FT, free testosterone;

DHEAS, dehydroepiandrosterone sulphate;

SHBG, sex hormone-binding globulin;

FAI, free androgen index;

HOMA-IR, homeostasis model assessment for insulin resistance;

IR, insulin resistance, HOMA≥ 2.14;

TC, total cholesterol;

TG, triglycerides;

HDL-C, high-density lipoprotein cholesterol;

LDL-C, low-density lipoprotein cholesterol.

*P<0.05,

**P<0.01, pre-treatment *vs* post-treatment.

## Discussion

To the best of our knowledge, this is the first study to evaluate the effect of berberine treatment alone on the menstrual pattern, ovulation rate, hormonal and metabolic profiles in anovulatory PCOS women. Women were classified into normal weight and overweight/obese groups in order to further evaluate the impact of weight on these effects.

Berberine, is recently found to have a potential glucose lowering effects on patients with diabetes and obesity in vitro and in vivo studies [13]. Ni *et al*. found that when berberine 0.3–0.5g, three times daily was administrated to 60 patients with type 2 diabetes for 1–3 months, fasting plasma glucose concentrations were reduced from 11.6mmol/L to 6.6 mmol/L [4]. Xie *et al*. reported that fasting and postprandial plasma glucose concentrations were reduced by 21% and 27%, respectively when treated with berberine 0.3–0.5g, three times daily [14]. Zhang *et al*. administered berberine 0.5g twice times daily for 3 months and found plasma glucose, TG, TC and LDL-C decreased significantly compared to placebo [15]. Wei *et al*. reported treatment with berberine plus oral contraceptives in 89 PCOS patients improved insulin resistance and lipid profile [16].

The mechanism of berberine in the treatment of diabetes remains unknown. Ko *et al*. found berberine enhanced insulin / insulin-like growth factor-1 signaling cascade and increased glucose-stimulated insulin secretion in Min6 cells [17]. Pan *et al*. reported berberine inhibited the activity of alpha-glucosidase and decreased glucose transport in Caco-2 cell [18]. Berberine is found to increase glucose uptake in HepG2 and 3T3-L1 cells, and enhances glucose metabolism by stimulation of glycolysis [19–21]. After four months of berberine, 14 women (14.3%) had regular menses and there is no difference between normal weight and overweight/obese groups. 51 of 98 subjects had at least 1 ovulatory cycle during the 4-month berberine treatment. The ovulation rate was 25.0% in the four-month treatment. The results indicated berberine alone has a mediocre impact on the menstrual pattern and the ovulation rate. We did not observe any difference in the menstrual pattern and the ovulation rate between normal weight and overweight/obese groups.

Metformin has been shown to be effective in the treatment of anovulation in PCOS women. Bridger *et al*. reported metformin could improve oligomenorrhea in a randomized placebo-controlled trial of metformin for adolescents with PCOS [22]. Palomba *et al*. conducted a 6-month randomized controlled clinical trial comparing clomiphene citrate (CC) and metformin for ovulation induction [12]. The ovulation rate was similar in metformin and CC groups (62.9% *vs* 67.0%). Legro *et al*. reported the ovulation rate was 29.0% in the metformin group, 49.0% in the CC group and 60.4% in the combination group of CC and metformin [23]. Zain *et al*. reported an ovulation rate of 23.7% in the metformin group, 59% in the CC group and 68.4% in the combination group [24]. A Cochrane review showed that metformin can improve the ovulation rate (OR 1.81; 95% CI 1.13–2.93) compared with placebo or no treatment but has a lower ovulation rate (OR 0.43; 95% CI 0.36–0.51) compared with CC [3]. In our study, the ovulation rate was 25.0% after 4 month berberine treatment in the whole group, 22.5% in the normal-weight group and 31.0% in the overweight/obese group. The ovulation rate of berberine in our study is similar to that of metformin in the study of Legro *et al*. and Zain *et al*. [22–23]. Our results showed berberine used alone could improve ovulation rate similar to metformin alone. Further studies are needed to compare the efficacy in ovulation induction between berberine, metformin, CC or combination.

The hormonal and metabolic characteristics were compared before and after berberine treatment. An *et al*. compared the efficacy of berberine, metformin or placebo in PCOS patients prior to IVF treatment [6]. SHBG increased in both berberine and metformin groups with decrease of TT and FAI. Wei *et al*. in a randomized study compared the effects of berberine on metabolic characteristics with metformin and placebo groups [16]. Oral conceptive (OC) including ethiniyl estradiol and cyproterone acetate was administrated simultaneously. An increase in SHBG was shown in both berberine and metformin groups. In our study, the SHBG level decreased after treatment in the whole group and such reduction was in the normal weight group only. Our results were contradictory to that of Wei’s study. The increase of SHBG in Wei’s study may be partly due to effect of OC. It is difficult to assess the effect of berberine on SHBG level when combined with OC. Legro *et al*. reported the metformin group had a significant decrease in TT and a significant increase in SHBG with a corresponding decrease in FAI [22]. Our results also suggested that berberine might have a different effect on SHBG compared with metformin. Decrease of SHBG and increase of FAI after berberine treatment showed berberine had no obvious efficacy on hyperandrogenism. Further studies are needed to prove the results and clarify the mechanism.

Mohiyiddeen *et al*. found a significant decrease of TG and TC in patients with PCOS after metformin treatment but the sample size was small [25]. Ladson *et al*. compared the effects of metformin with lifestyle therapy in a randomized double-blind study [26]. TC, TG and LDL-C increased, but it had no significant difference. HDL-C decreased significantly in both metformin and lifestyle groups, showing the effect of lifestyle modification. Wei *et al*. compared the short-term effect of berberine with metformin on the metabolic features of PCOS patients. Increase of TC, TG, LDL-C and decrease of HDL-C were found in berberine group [16]. Only decrease of HDL-C was observed in metformin group. An *et al*. reported a decrease of TG and LDL-C in both berberine and metformin groups, which was more significant in the berberine group [6]. In our study, TC, TG and LDL-C decreased after treatment in the normal-weight group only. Only TC decreased in the overweight/obese group after treatment. Our results were similar to Wei’ study [16]. It is interesting that the improvement of lipid profile is significant in the normal weight group only. We confirmed an improvement of IR in the normal weight group only after berberine treatment.

In conclusion, our study found that berberine alone may improve the menstrual pattern and the ovulation rate in anovulatory Chinese women with polycystic ovary syndrome. Berberine can decrease sex hormone binding globulin, insulin resistance, total cholesterol, triglycerides and low-density lipoprotein cholesterol in normal weight patients with PCOS.

### Limitations

There was no placebo or active treatment for comparison in our pilot pre- and post-treatment study. However, we feel it is important to have information regarding the ovulation rate following berberine which can be used for sample size calculation in subsequent randomized trials comparing the effects between berberine and CC or metformin. The other limitation is the lack of the pregnancy rate as it is still not clear if berberine may cause any congenital abnormalities. Therefore, subjects were advised to use contraception during the study period.

## Supporting Information

S1 File Study protocol submitted to the ethics committee of Sun Yat-sen Memorial Hospital (Chinese version). (DOC)

S2 File Study protocol submitted to the ethics committee of Sun Yat-sen Memorial Hospital (English version). (DOC)

S3 File CONSORT checklist. (DOC)

S4 File TREND checklist. (PDF)

## References

[1] The Rotterdam ESHRE/ASRM-sponsored PCOS consensus workshop group (2004) Revised 2003 consensus on diagnostic criteria and long term health risks related to polycystic ovary syndrome (PCOS). Hum Reprod 19: 41–47. 14688154 10.1093/humrep/deh098

[2] Angioni S, Portoghese E, Milano F, Melis GB, Fulghesu AM (2008) Diagnosis of metabolic disorders in women with polycystic ovary syndrome. Obstet Gynecol Surv 63: 796–802. 10.1097/OGX.0b013e3181895a06 19017415

[3] Tang T, Lord JM, Norman RJ, Yasmin E, Balen AH (2012) Insulin-sensitising drugs (metformin, rosiglitazone, pioglitazone, D-chiro-inositol) for women with polycystic ovary syndrome, oligo amenorrhoea and subfertility. Cochrane Database Syst Rev 5: CD003053 10.1002/14651858.CD003053.pub5 22592687

[4] Ni YX (1998) Therapeutic effect of berberine on 60 patients with type II diabetes mellitus and experimental research. Zhong Xi Yi Jie He Za Zhi 8: 711–713. [article in Chinese] 3248329

[5] Li Y, Kuang H, Shen W, Ma H, Zhang Y, Stener-Victor E, et al (2013) Letrozole, berberine, or their combination for anovulatory infertility in women with polycystic ovary syndrome: study design of a double-blind randomized controlled trial. BMJ Open 3: e003934 10.1136/bmjopen-2013-003934 24282248 PMC3845065

[6] An Y, Sun Z, Zhang Y, Liu B, Guan Y, Lu M (2014) The use of berberine for women with polycystic ovary syndrome undergoing IVF treatment. Clin Endocrinol 80: 425–431. 10.1111/cen.12294 23869585

[7] The World Health Organization Western Pacific Region. Redefining obesity and its treatment In: The International Association for the Study of Obesity, and the International Obesity Task Force: The Asia-Pacific Perspective. Melbourne: Health Communication Australia; 2000 pp. 15–21.

[8] Azziz R, Woods KS, Reyna R, Key TJ, Knochenhauer ES, Yildiz BO (2004) The prevalence and features of the polycystic ovary syndrome in an unselected population. J Clin Endocrinol Metab 89: 2745–2749. 15181052 10.1210/jc.2003-032046

[9] Palomba S, Orio F Jr, Falbo A, Manguso F, Russo T, Casella T, et al (2005) Prospective parallel randomized, double-blind, double-dummy controlled clinical trial comparing clomiphene citrate and metformin as the first-lin treatment for ovulation induction in nonobese anovulatory women with polycystic ovary syndrome. J Clin Endocrinol Metab 90: 4068–4074. 15840746 10.1210/jc.2005-0110

[10] Zhao X, Ni R, Li L, Mo Y, Huang J, Huang M, et al (2011) Defining hirsutism in Chinese women: a cross-sectional study. Fertil Steril 96: 792–796. 10.1016/j.fertnstert.2011.06.040 21762890

[11] Mathur RS, Moody LO, Landgrebe S, Willianson HO (1981) Plasma androgens and sex hormone binding globulin in the evaluation of hirsute patients. Fertil Steril 35: 29–35. 6450691 10.1016/s0015-0282(16)45254-4

[12] Chen X, Yang D, Li L, Feng S, Wang L (2006) Abnormal glucose tolerance in Chinese women with polycystic ovary syndrome. Hum Reprod 21: 2027–2032. 16684838 10.1093/humrep/del142

[13] Gu Y, Zhang Y, Shi X, Li X, Hong J, Chen J, et al (2010) Effect of traditional Chinese medicine berberine on type 2 diabetes based on comprehensive metabonomics. Talanta 81: 766–772. 10.1016/j.talanta.2010.01.015 20298851

[14] Xie P, Zhou H, Gao Y (2005) The clinical efficacy of berberine in treatment of type 2 diabetes mellitus. Chinese Journal of Clinical Healthcare 8: 402–403. [article in Chinese]

[15] Zhang Y, Li X, Zou D, Liu W, Yang J, Zhu N, et al (2008) Treatment of type 2 diabetes and dyslipidemia with the natural plant alkaloid berberine. J Clin Endocrinol Metab 93: 2559–2565. 10.1210/jc.2007-2404 18397984

[16] Wei W, Zhao H, Wang A, Sui M, Liang K, Deng H, et al (2012) A clinical study on the short-term effect of berberine in comparison to metformin on the metabolic characteristics of women with polycystic ovary syndrome. Eur J Endocrinol 166: 99–105. 10.1530/EJE-11-0616 22019891

[17] Ko BS, Choi SB, Park SK, Jang JS, Kim YE, Park S(2005) Insulin sensitizing and insulinotropic action of berberine from Cortidis rhizoma. Biol Pharm Bull 28: 1431–1437. 16079488 10.1248/bpb.28.1431

[18] Pan GY, Huang ZJ, Wang GJ, Fawcett JP, Liu XD, Zhao XC, et al (2003) The antihyperglycaemic activity of berberine arises from a decrease of glucose absorption. Planta Med 69: 632–636. 12898419 10.1055/s-2003-41121

[19] Yin J, Hu R, Chen M, Tang J, Li F, Yang Y, et al (2002) Effects of berberine on glucose metabolism in vitro. Metabolism 51: 1439–1443. 12404195 10.1053/meta.2002.34715

[20] Zhou L, Yang Y, Wang X, Liu S, Shang W, Yuan G, et al (2007) Berberine stimulates glucose transport through a mechanism distinct from insulin. Metabolism 56: 405–412. 17292731 10.1016/j.metabol.2006.10.025

[21] Yin J, Gao Z, Liu D, Liu Z, Ye J (2008) Berberine improves glucose metabolism through induction of glycolysis. Am J Physiol Endocrinol Metab 294: E148–156. 17971514 10.1152/ajpendo.00211.2007 PMC2464622

[22] Bridger T, MacDonald S, Baltzer F, Rodd C (2006) Randomized placebo-controlled trial of metformin for adolescents with polycystic ovary syndrome. Arch Pediatr Adolesc Med 160: 241–246. 16520442 10.1001/archpedi.160.3.241

[23] Legro RS, Barnhart HX, Schlaff WD, Carr BR, Diamond MP, Carson SA, et al (2007) Clomiphene, metformin, or both for infertility in the polycystic ovary syndrome. N Eng J Med 356: 551–566. 10.1056/NEJMoa063971 17287476

[24] Zain MM, Jamaluddin R, Ibrahim A, Norman RJ (2009) Comparison of clomiphene citrate, metformin, or the combination of both for first-lin ovulation induction, achievement of pregnancy, and live birth in Asian women with polycystic ovary syndrome: a randomized controlled trial. Fertil Steril 91: 514–521. 10.1016/j.fertnstert.2007.12.002 18321486

[25] Mohlyiddeen L, Watson AJ, Apostolopoulos NV, Berry R, Alexandraki KI, Jude EB (2013) Effects of low-dose metformin and rosiglitazone on biochemical, clinical, metabolic and biophysical outcomes in polycystic ovary syndrome. J Obstet Gynaecol 33: 165–170. 10.3109/01443615.2012.745839 23445141

[26] Ladson G, Dodson Wc, Sweet SD, Archibong AE, Kunselman AR, Demers LM, et al (2011) The effects of metformin with lifestyle therapy in polycystic ovary syndrome: a randomized double-blind study. Fertil Steril 95: 1059–1066. e 1–7. 10.1016/j.fertnstert.2010.12.002 21193187 PMC3073705

