# Supplementary material for: A Single Arm Pilot Study of Effects of Berberine on the Menstrual Pattern, Ovulation Rate, Hormonal and Metabolic Profiles in Anovulatory Chinese Women with Polycystic Ovary Syndrome
Source: PLoS One. 2015 Dec 8;10(12):e0144072. doi: 10.1371/journal.pone.0144072 (PMC4672885; doi:10.1371/journal.pone.0144072)
Supplement: S1 File — (DOC) [file pone.0144072.s001.doc]

**黄连素对中国多囊卵巢综合征患者月经和代谢影响的前瞻性研究**

一、研究背景

多囊卵巢综合征是生育年龄妇女最常见的内分泌疾病，以胰岛素抵抗、高雄激素血症和排卵障碍为主要临床特征，具有高度异质性。2003年鹿特丹ESHER/ASRM会议制定了多囊卵巢综合征(Polycystic ovary syndrome, PCOS)的诊断标准：偶发排卵或无排卵，临床和/或生化高雄激素血症，卵巢多囊性改变，符合其中二项并排除其它病因。目前对高雄激素是否应做为必要诊断标准还存在争议，AES强调必须有高雄激素表现才能诊断PCOS。

PCOS患者代谢异常的发病率增高，在不同人种约为15%-40%，广州和香港人群的发病率分别报道为17%和25%，主要表现为胰岛素抵抗，常用空腹血糖胰岛素比值(GIR)、HOMA和QUICKI评估胰岛素抵抗程度。胰岛素增敏剂可以增加靶组织的胰岛素敏感性，降低代偿性高胰岛素血症，并逆转胰岛素抵抗对排卵功能的负面影响。二甲双胍是最常用的胰岛素增敏剂，常用剂量是500mg tid或850mg bid，可明显提高排卵率和临床妊娠率，联合使用二甲双胍和克罗米芬有助于提高排卵率和临床妊娠率，但并不影响活产率。双胍类药物最常见的副作用是胃肠道反应，如恶心，呕吐，腹泻，严重副作用乳酸酸中毒较罕见，因此肝肾功能异常，严重心血管疾病或缺氧的病人不宜使用。

黄连素是从中草药黄连中提取出来的一种天然植物生物碱，常用于腹泻治疗。近年来多项研究发现黄连素可用于治疗糖尿病和肥胖，在db/db小鼠和高脂饮食Wistar小鼠中改善葡萄糖代谢异常，在 Min6细胞中增加葡萄糖刺激的胰岛素分泌，抑制α葡萄糖苷酶活性，在Caco-2细胞中降低葡萄糖吸收，在HepG2和3T3-L1细胞中促进葡萄糖摄入并不受胰岛素分泌影响，通过糖酵解改善葡萄糖代谢，在HIT-T15细胞、小鼠胰岛细胞和BALB/C大鼠中促进胰岛素分泌，上述研究均提示黄连素在体内外研究中可改善胰岛素抵抗和糖代谢异常。

近年来发现黄连素在临床上可直接用于糖尿病治疗。60名2型糖尿病患者在每天3次服用黄连素0.3-0.5g，1-3个月后空腹血糖从11.6mmol/L降至6.6mmol/L。40名2型糖尿病患者在原来治疗上加用每天3次黄连素0.3-0.5g，2个月后空腹和餐后血糖分别下降21%和27%。另一研究结果显示30名有脂肪肝的2型糖尿病患者每天3次服用黄连素0.5g，2个月后血糖、甘油三酯和总胆固醇分别降低31%、40%和23%，并伴有谷丙转氨酶和谷草转氨酶的降低。上述研究中除1例患者有轻度胃肠道不适外，其余患者均能很好耐受黄连素。

PCOS患者中代谢异常发生率较高，尤其是肥胖患者，目前广泛应用的胰岛素增敏剂二甲双胍因胃肠道反应常见而影响患者的耐受性和依从性。黄连素是一种安全有效的中药制剂，已有研究报道可用于糖尿病治疗，我们推测黄连素有可能改善PCOS的胰岛素抵抗和代谢异常，有助于减轻副作用而提高患者的耐受性和依从性。目前尚未有关于黄连素用于治疗PCOS患者月经和代谢异常的研究报道。因此，本研究拟将黄连素用于PCOS患者治疗，观察其对月经和代谢异常的影响。

二、研究目的

1. 评估中国PCOS患者黄连素治疗4个月的有效性，观察其对月经模式、排卵率、内分泌和代谢指标的影响。
2. 评估中国PCOS患者黄连素治疗的安全性，观察记录治疗期间的副作用。
3. 评估中国PCOS患者黄连素治疗停药4个月的有效性，观察其对月经模式、排卵率、内分泌和代谢指标的后续影响。

三、研究方法

1．方法：前瞻性观察研究。

2．研究对象：

中山大学孙逸仙纪念医院生殖中心就诊的中国PCOS患者。

纳入标准：①年龄18-40岁；②符合Rotterdam标准；③月经稀发或无排卵；④1年内不计划怀孕或采用避孕套避孕。

排除标准：①月经规则；②3个月内曾服用激素治疗。

1. 分组：

根据患者体重分为两组：①正常体重组：BMI≤23Kg/m^2^，40例；②超重/肥胖组：BMI＞23Kg/m^2^，40例。

4．治疗方案：

所有患者均服用4个月黄连素，0.4g，每天3次。

5．筛查指标

⑴ 一般临床资料：月经情况，血压，身高，体重，腰围，臀围，改良Ferriman-Gallwey多毛评分。

⑵ B超检查：所有参与者排空膀胱后用5-9MHz腹部/阴道探头进行经阴道或直肠超声检查。

⑶激素测定：在早卵泡期(自然月经周期或黄体酮撤退性出血第2-5天)空腹进行抽血，标本检测FSH、LH、总睾酮、SHBG、雄烯二酮、DHEAS、血糖、胰岛素和血脂，并进行葡萄糖耐量试验。每周测定孕酮水平。

⑷ 在治疗前和治疗结束后填写生活质量量表（SF-36和PCOSQOL)。

⑸ 记录月经情况，副作用。

6. 随访；治疗前到治疗结束后4个月，每月1次，监测指标同上。

7．统计学

⑴ 样本量计算

作为一个前瞻性研究，在此领域没有相关数据。计划招募40例肥胖和40例体重正常的多囊卵巢综合征患者。

⑵ 统计方法

主要指标包括一般临床资料（血压，体重，腰围，臀围）、月经与排卵情况、激素水平（FSH、LH、总睾酮、SHBG、雄烯二酮、DHEAS、血糖、胰岛素和血脂）。正态分布的数据用均数±标准差表示，非正态分布的数据用中位数表示。治疗前后的指标比较采用Student’s t检验，Mann-Whitney U-test，Wilcoxon signed-rank检验。以P<0.05为有统计学意义。

四、 实验进程图

就诊病人（纳入标准、排除标准），分组

进入黄连素治疗周期

观测指标（月经情况、激素水平、卵巢容积、窦卵泡数等）

从治疗4个月到治疗结束后4个月，每月随访

数据统计分析
