# Supplementary material for: A Single Arm Pilot Study of Effects of Berberine on the Menstrual Pattern, Ovulation Rate, Hormonal and Metabolic Profiles in Anovulatory Chinese Women with Polycystic Ovary Syndrome
Source: PLoS One. 2015 Dec 8;10(12):e0144072. doi: 10.1371/journal.pone.0144072 (PMC4672885; doi:10.1371/journal.pone.0144072)
Supplement: S2 File — (DOC) [file pone.0144072.s002.doc]

**TITLE**

A prospective study of the effect of Berberine taken for 4 months in Chinese women with polycystic ovary syndrome on the menstrual pattern, ovulation rate, hormonal and metabolic profiles

**INVESTIGATORS**

Professor Yang Dongzi

Dr. Ng Hung Yu Ernest

Professor Wu Xiaoke

**BACKGROUND INFORMATION**

Polycystic ovary syndrome (PCOS) is one of the commonest disorders in reproductive endocrinology among women in the reproductive age. It is a heterogeneous condition characterized pathophysiologically by insulin resistance, hyper- androgenism and gonadotrophic disturbances leading to oligoanovulation.

Since the first report by Stein and Levanthal in 1930, different combinations of clinical, biochemical and sonographical parameters have been used to define this condition with little international consensus for many decades. It was until 2003 that a consensus meeting at Rotterdam agreed on the diagnostic criteria which consisted of the presence of two out of the following three: oligo- and/or anovulation, clinical and/or biochemical hyperandrogenism, and ultrasound features of polycystic ovaries, with the exclusion of other aetiologies (Rotterdam ESHRE/ASRM-Sponsored PCOS Consensus Workshop Group, 2004). Polycystic ovaries is defined by transvaginal ultrasound scan showing at least 12 follicles between 2 to 9 mm in diameter or an ovarian volume of more than 10 ml in either ovary (Balen et al, 2003). It has since become the most widely adopted diagnostic criteria contributing essentially to the standardization for research and clinical practice purposes.

However, there have still been controversies on whether androgen excess (AE) should be an essential feature of PCOS. Most recently, the Androgen Excess and PCOS Society laid down the recommendation in their task force report that the diagnosis of PCOS should not be established without evidence of either clinical or biochemical hyperandrogenism, although the exact measures for these may vary (Azziz et al, 2009). The essence of the AE-PCOS Society criteria relies heavily on the relationship of AE with metabolic dysfunction (Azziz et al, 2009), which is another long-term concern for PCOS patients. Metabolic syndrome has been found more prevalent among PCOS patients compared to controls, and the prevalence varied from about 15% up to over 40% in different ethnicities, with rates of 17% (Ni et al, 2009) and 25% (Cheung et al, 2008) being recently reported among PCOS patients in Guangzhou and Hong Kong respectively.

In women with PCOS, insulin resistance is one recognized metabolic disturbance. Fasting glucose:insulin ratio (GIR), homeostatic model assessment of insulin resistance (HOMA-IR) and quantitative insulin sensitivity check index (QUICKI) are more commonly used indices of insulin resistance (Angioni et al, 2008). Insulin-sensitising agents can increase the insulin responsiveness in target tissues, reduce the compensatory hyperinsulinaemia and hence ameliorating its adverse effect on ovulatory function.

Metformin, a biguanide, is one of the most commonly used insulin sensitiser and can be given at 500 mg three times daily or 850 mg two times daily with meals. As reported in a Cochrane review (Tang et al., 2010), metformin used alone improves the ovulation rate (OR 2.12; 95% CI 1.5–3.0) and clinical pregnancy rate (OR 3.86; 95% CI 2.18–6.84) compared with placebo or no treatment, but not the livebirth rate (OR 1.0; 95% CI 0.16–6.39). When compared with clomiphene citrate (CC), metformin leads to lower ovulation rate (OR 0.48; 95% CI 0.41–0.57) and clinical pregnancy rate (OR 0.63; 95% CI 0.43–0.92), and a non-significant trend of lower livebirth rate (OR 0.67; 95% CI 0.44–1.02). Co-treatment with metformin and CC improves the ovulation rate (OR 1.76; 95% CI 1.51–2.06) and clinical pregnancy rate (OR 1.48; 95% CI 1.12–1.95), but not the livebirth rate (OR 1.05; 95% CI 0.75–1.47) compared with CC alone.

Gastrointestinal upset including nausea, vomiting, diarrhea are the most common side effects. Lactic acidosis is a rare though serious complication, and hence it is contraindicated in patients with renal, hepatic or major cardiovascular disease or hypoxia.

Berberine, a natural plant alkaloid isolated from the Chinese herb, Coptis Chinensis (Huanglian), is commonly used for diarrhea, and a potential glucose lowering effect has been noted (Ni, 1998). In vitro and in vivo studies subsequently showed that berberine has potentially beneficial effects in the treatment of diabetes and obesity. Berberine can reduce body weight and cause a significant improvement in glucose tolerance in db/db mice and high-fat-fed Wistar rats (Lee et al., 2006). Berberine may increase glucose-stimulated insulin secretion and proliferation in Min6 cells (Ko et al., 2005), and inhibit α-glucosidase activities and reduce glucose absorption in Caco-2 cell (Pan et al., 2003). Berberine promoted glucose uptake in HepG2 and 3T3-L1 cells independent of insulin action (Yin et al., 2002; Zhou et al., 2007) and improved glucose metabolism via glycolysis (Yin et al., 2008). Berberine also facilitated insulin secretion in HIT-T15 cells and in murine pancreatic islets in vitro and in BALB/C mice (Leng et al., 2004).

Several clinical investigations of berberine in the treatment of diabetes were reported in Chinese over the past two decades. Ni et. al. (1988) reported that fasting plasma glucose concentrations in 60 patients with type 2 diabetes were reduced from 11.6mmol/L to 6.6 mmol/L for 1-3 months when treated with berberine (0.3-0.5g, three times daily). Xie et. al. (2005) found that when berberine (0.3-0.5g, three times daily) was administrated to 40 type 2 diabetic patients for 2 months without change in their previous therapy, fasting and postprandial plasma glucose concentrations were reduced by 21% and 27%, respectively. Wei et. al. (2004) also reported that treatment with berberine (0.5g, three times daily) for 2 months in 30 type 2 diabetic patients with fatty liver decreased fasting plasma glucose, triglyceride and total cholesterol concentrations by 31%, 40% and 23%, respectively, and was associated with decrease in serum alanine aminotransferase and aspartate aminotransaminase concentrations. Participants in these clinical studies tolerated berberine well, with one subject in one study having mild gastrointestinal discomfort.

Berberine may be used in patients with PCOS and may improve ovulation rate and pregnancy rate following improvement of insulin resistance. There is still no published study on the use of berberine on the menstrual pattern and ovulatory rate of women with PCOS.

References:

1. Angioni S, Portoghese E, Milano F, Melis GB, Fulghesu AM. Diagnosis of metabolic disorders in women with polycystic ovary syndrome. Obstet Gynecol Surv 2008; 63(12): 796-802.
2. Azziz R, Carmina E, Dewailly D, Diamanti-Kandarakis E, Escobar-Morreale HF, Futterweit W, Janssen OE, Legro RS, Norman RJ, Taylor AE, Witchel SF. The Androgen Excess and PCOS Society criteria for the polycystic ovary syndrome: the complete task force report. Fertil Steril 2009; 91(2): 456-488.
3. Balen AH, Laven JS, Tan SL, Dewailly D. Ultrasound assessment of the polycystic ovary: international consensus definition. Hum Reprod Update 2003; 9:505-514.
4. Cheung LP, Ma RCW, Lam PM, Lok IH, Haines CJ, So WY, Tong PCY, Cockram CS, Chow CC, Goggins WB. Cardiovascular risks and metabolic syndrome in Hong Kong Chinese women with polycystic ovary syndrome. Hum Reprod 2008; 23(6): 1431-1438.
5. Ko BS, Choi SB, Park SK, Jang JS, Kim YE, Park S 2005. Insulin sensitizing and insulinotropic action of berberine form cortidis rhizoma. Biol Pharm Bull 28:1431-1437
6. Lee YS, Kim WS, Kim KH, Yoon MJ, Cho HJ, Shen Y, Ye JM, Lee CH, Oh WK, Kim CT, Hohnen-Behrens C, Gosby A, Kraegen EW, James DE, Kim JB 2006. Berberine, a natural plant product, activates AMP-activated protein kinase with beneficial metabolic effects in diabetic and insulin-resistance states. Diabetes 55:2256-2264
7. Leng SH, Lu FE, Xu LJ 2004. Therapeutic effects of berberine in impaired glucose tolerance rats and its influence on insulin secretion. Acta Pharmacol Sin 25:496 –502
8. Ni YX 1988. Therapeutic effect of berberine on 60 patients with type II diabetes mellitus and experimental research. Zhong Xi Yi Jie He Za Zhi 8:711–713 [article in Chinese]
9. Ni RM, Mo Y, Chen X, Zhong J, Liu W, Yang D. Low prevalence of the metabolic syndrome but high occurrence of various metabolic disorders in Chinese women with polycystic ovary syndrome. Eur J Endocrinol 2009; 161: 411-418.
10. Pan GY, Huang ZJ, Wang GJ, Fawcett JP, Liu XD, Zhao XC, Sun JG, Xie YY 2003. The antihyperglycaemic activity of berberine arises from a decrease of glucose absorption. Planta Med 69:632-636
11. Tang T, Lord JM, Norman RJ, Yasmin E, Balen AH. Insulin-sensitising drugs (metformin, rosiglitazone, pioglitazone, D-chiro-inositol) for women with polycystic ovary syndrome, oligo amenorrhoea and subfertility. Cochrane Database Syst Rev 2010;Issue 1:CD003053.
12. The Rotterdam ESHRE/ASRM-sponsored PCOS consensus workshop group. Revised 2003 consensus on diagnostic criteria and long term health risks related to polycystic ovary syndrome (PCOS). Hum Reprod 2004; 19: 41-47.
13. Wei J, Wu J, Jiang J, Wang S, Wang Z 2004. Clinical study on improvement of type 2 diabetes mellitus complicated with fatty liver treatment by berberine. Zhong Xi Yi Jie He Ganbing Za Zhi 14:334-336 [article in Chinese]
14. Xie P, Zhou H, Gao Y 2005. The clinical efficacy of berberine in treatment of type 2 diabetes mellitus. Chinese Journal of Clinical Healthcare 8:402-403 [article in Chinese]
15. Yin J, Hu R, Chen M, Tang J, Li F, Yang Y, Chen J 2002. Effects of berberine on glucose metabolism in vitro. Metabolism 51:1439-1443
16. Yin J, Gao Z, Liu D, Liu Z, Ye J 2008. Berberine improves glucose metabolism through induction of glycolysis. Am J Physiol Endocrinol Metab 294:E148-156.
17. Zhou L, Yang Y, Wang X, Liu S, Shang W, Yuan G, Li F, Tang J, Chen M, Chen J 2007. Berberine stimulates glucose transport through a mechanism distinct from insulin. Metabolism 56:405-412

**OBJECTIVES**

1. To evaluate the effect of berberine taken for 4 months in Chinese women with PCOS on the menstrual pattern, ovulation rate, hormonal and metabolic profiles。
2. To evaluate the side effects in Chinese women with PCOS who are taking berberine for 4 months.
3. To document the changes on the menstrual pattern, ovulation rate, hormonal and metabolic profiles in Chinese women with PCOS over another 4 months after stopping berberine.

**METHODOLOGY**

1. Study design: This is a prospective observation study.
2. Participants are recruited in the outpatients of Gynecology of Sun Yat-sen Memorial Hospital.

Inclusion criteria:

1. Chinese;
2. Age >18 and <40 years;
3. PCOS women according to Rotterdam criteria;
4. Oligomenorrhea or anovulation;
5. No fertility wish within one year or using male condoms.

Exclusion criteria:

1. Women with regular cycles;
2. On hormonal preparation within 3 months.
3. Two groups
4. BMI ≤ 23 kg/m^2^ (normal weight group): 40 women to be recruited;
5. BMI > 23 kg/m^2^ (overweight / obese group): 40 women to be recruited.
6. Treatment

Berberine 0.4g three times daily for 4 months.

1. Screening and monitoring
2. The following clinical data will be collected: blood pressure, body height, body weight, waist circumference, hip circumference, and the modified Ferriman-Gallwey hirsutism score.
3. After an overnight fast, the subjects will attend the clinic for a transvaginal or transrectal ultrasound examination and blood test in the early follicular phase (Day 2-5) of a spontaneous period in women with regular periods or a withdrawal bleeding in women with long irregular cycles. Ultrasound examinations will be performed at 8–10 a.m. with a 5-9 MHz abdominal / transvaginal probe, after the patient has emptied the bladder.
4. Fasting blood will then be taken for the measurement of FSH, LH, total testosterone, SHBG, androstenedione, dehydroepiandrosterone sulphate (DHEAS), glucose and insulin concentrations, and lipid profile. Oral glucose tolerance test is done. Weekly serum progesterone concentration is monitored.
5. The subjects are asked to complete quality of life questionnaire (SF-36 and PCOSQOL) at screening, 4 months after berberine.
6. Menstrual calendar, side effect diary are recorded monthly.
7. Follow up
8. Every month;
9. Measure blood pressure, body weight, waist circumference, hip circumference;
10. USS for ovarian morphology and size of the ovary;
11. Repeat hormonal and metabolic parameters;
12. Review side effect;
13. Follow up continued for 4 months after stopping berberine.
14. Statistical analysis
15. Sample size calculation

This is the pilot study comparing the pre-treatment and post-treatment of berberine in PCOS patients. We need to have the results of the pilot study regarding ovulation rate which can be used for sample size calculation in subsequent randomized trials. We plan to recruit 40 obese and 40 normal-weight participants.

1. Statistical methods

Continuous variables were given as mean ± standard deviation (SD) if normally distributed, and as median (range) if not normally distributed. Statistical comparison was carried out by Student’s T test, Mann-Whitney U-test, Wilcoxon signed ranks test for continuous variables. The two-tailed value of *P* < 0.05 was considered statistically significant.

**FLOW CHART**

Recruitment and grouping

Berberine treatment

Monitoring indexes (menstrual cycles, ovulation, hormones)

Follow up monthly 4 months after stopping berberine

Data analysis
